# A Pyramid Deep Feature Extraction Model for the Automatic Classification of Upper Extremity Fractures

**DOI:** 10.3390/diagnostics13213317

**Published:** 2023-10-26

**Authors:** Oğuz Kaya, Burak Taşcı

**Affiliations:** 1 Department of Orthopedics and Traumatology, Elazig Fethi Sekin City Hospital, Elazig 23280, Turkey; 2 Vocational School of Technical Sciences, Firat University, Elazig 23119, Turkey

**Keywords:** musculoskeletal radiographs, Efficientb0, upper extremity, pyramid model, SVM, NCA

## Abstract

The musculoskeletal system plays a crucial role in our daily lives, and the accurate diagnosis of musculoskeletal issues is essential for providing effective healthcare. However, the classification of musculoskeletal system radiographs is a complex task, requiring both accuracy and efficiency. This study addresses this challenge by introducing and evaluating a pyramid deep feature extraction model for the automatic classification of musculoskeletal system radiographs. The primary goal of this research is to develop a reliable and efficient solution to classify different upper extremity regions in musculoskeletal radiographs. To achieve this goal, we conducted an end-to-end training process using a pre-trained EfficientNet B0 convolutional neural network (CNN) model. This model was trained on a dataset of radiographic images that were divided into patches of various sizes, including 224 × 224, 112 × 112, 56 × 56, and 28 × 28. From the trained CNN model, we extracted a total of 85,000 features. These features were subsequently subjected to selection using the neighborhood component analysis (NCA) feature selection algorithm and then classified using a support vector machine (SVM). The results of our experiments are highly promising. The proposed model successfully classified various upper extremity regions with high accuracy rates: 92.04% for the elbow region, 91.19% for the finger region, 92.11% for the forearm region, 91.34% for the hand region, 91.35% for the humerus region, 89.49% for the shoulder region, and 92.63% for the wrist region. These results demonstrate the effectiveness of our deep feature extraction model as a potential auxiliary tool in the automatic analysis of musculoskeletal system radiographs. By automating the classification of musculoskeletal radiographs, our model has the potential to significantly accelerate clinical diagnostic processes and provide more precise results. This advancement in medical imaging technology can ultimately lead to better healthcare services for patients. However, future studies are crucial to further refine and test the model for practical clinical applications, ensuring that it integrates seamlessly into medical diagnosis and treatment processes, thus improving the overall quality of healthcare services.

## 1. Introduction

Bones, as a fundamental component of the human skeletal system, are metabolically active and structurally dynamic [1]. They also play significant roles in the metabolic, endocrine, and hematological systems while providing a mineral-containing connective tissue that supports the vital organs of the body and enables the mobility of the skeletal system [2,3]. Bones play a crucial role in transforming the forces generated by muscle contractions into body movements through a lever system [4]. A fracture refers to a condition in which the integrity of bones is disrupted due to internal or external factors [5]. Fractures not only affect bones but can also impact surrounding tissues and lead to systemic complications, making them a general traumatological event [6]. The healing of bone fractures involves a complex series of cellular and molecular events, including a specific wound-healing process [7]. Bone is one of the rare tissues that can heal without forming fibrous scar tissue, and the healing process can occur directly or indirectly [8]. Bone fractures are among the most common reasons for hospital admissions, especially in cases of high-energy trauma such as falls from heights or traffic accidents [9]. Factors like advanced age and osteoporosis can increase the risk of fractures, making them more prevalent in the elderly population [10]. This situation can elevate the cost of fracture treatment and extend the healing process. Therefore, the treatment of these patients becomes a significant issue in terms of both cost and effectiveness [11,12].

Today, artificial intelligence, which has made significant advances in the field of medicine, has also had noteworthy effects on the healthcare sector. One of these effects is the advancements in the diagnosis and treatment of orthopedic traumas such as bone fractures. Artificial intelligence algorithms, with their ability to rapidly and accurately analyze information obtained from radiological images, assist in making more precise and early diagnoses related to bone fractures. In the literature, numerous studies have been conducted in the fields of orthopedics and artificial intelligence. Some of these studies are provided below. In this manner, it becomes possible to alleviate the workload of healthcare professionals, utilize time more efficiently, and enhance diagnostic accuracy [13,14,15]. The development of computer-aided diagnostic systems holds particular significance for less developed and developing countries where there is an inadequacy of specialized experts.

Studies in the literature addressing the detection of orthopedic abnormalities using deep learning are enumerated below.

Sezer and colleagues [16] utilized a total of 219 shoulder bone MR images, comprising 91 edematous cases, 49 Hill–Sachs lesions, and 79 normal cases. Texture information obtained through the gray level co-occurrence matrix (GLCM) algorithm was combined with features obtained using gradient histogram pyramid algorithms for classification purposes. In these classification processes, a kernel-based support vector machine (SVM) achieved an 88% success rate. Additionally, by employing extreme learning machines (ELM), a 94% success rate was achieved. Wu and colleagues [17] examined the results obtained by using the feature ambiguity reduction operator (FAMO) model, which is employed in bone fracture detection, in conjunction with a 101-layer ResNeXt and feature pyramid network (FPN) on a dataset containing 9040 radiographic images. This study determined an average precision value of 77.40% for fracture detection. Ma and colleagues [18] utilized a dataset comprising 1052 bone images, of which 526 were fractured and the remainder were intact. The images were analyzed using Faster R-CNN and, as a result of this analysis, a crack-sensitive convolutional neural network (CrackNet) model achieved an accuracy of 90.11% for fracture detection on the entire dataset. Gan and colleagues [19] performed fracture detection by determining the position of the distal radius using anteroposterior wrist X-ray images. They developed a system for this purpose using Faster R-CNN and Inception-v4 architectures. The dataset consisted of 2340 images, with training and test sets containing 2040 and 300 images, respectively. The system’s performance with Faster R-CNN was evaluated at 0.87 based on the IoU success criterion. Inception-v4 was assessed based on metrics such as overall accuracy, sensitivity, specificity, Youden’s index, and AUC score, achieving classifier performances of 93%, 90%, 96%, 0.86, and 0.96, respectively. Furthermore, the system was compared with radiologists and orthopedic specialists, with radiologists demonstrating superior performance. Sezer and colleagues [20] proposed a computer-aided diagnosis (CAD) system based on Capsule Network (CapsNet) for the diagnosis of rotator cuff lesions in shoulder MR images. In this study, traditional methods such as CNN, AlexNet, GoogLeNet, and gray level co-occurrence matrix (GLCM) achieved overall accuracy rates of 93.21%, 88.45%, 87.63%, and 85.20%, respectively. The recommended CapsNet model outperformed these models, achieving an accuracy rate of 94.75%. On the other hand, Beyaz et al. [21] aimed to develop a convolutional neural network (CNN) model for classifying fractured and non-fractured femoral necks in frontal pelvic X-ray images. They curated a dataset consisting of 234 images from 65 patients and augmented it to 2106 images. Of these, 1341 were fractured femoral necks and 765 were non-fractured. The CNN architecture included five blocks with batch normalization, ReLU activation, and dropout layers, followed by a softmax classification layer. Training employed an Adam Optimizer, a learning rate schedule, and regularization to mitigate overfitting. The model was trained with various image resolutions (50 × 50, 100 × 100, 200 × 200, and 400 × 400) and hyperparameters were optimized using a genetic algorithm. An accuracy of 79.30% was achieved with the proposed method. Tobler and colleagues [22] investigated the performance of a deep convolutional neural network (DCNN) in detecting and classifying distal radius fractures, metal objects, and casts in radiographs using report-based labels. Their study included 15,775 radiographs and utilized a ResNet18 DCNN. With the 18-layer ResNet (ResNet-18) model, fracture detection achieved an accuracy of 94%. Tanzi and others [23] proposed a deep learning-based tool aimed at improving the diagnosis of bone fractures, with a focus on AO/OTA classification. The research used a large dataset consisting of proximal femur images and employed a multi-stage CNN architecture with InceptionV3 CNN. Image interpretation was performed using Grad-CAM, and the CNN’s performance was evaluated using various metrics. In fracture classification tasks, the InceptionV3 model achieved an accuracy of 87% for three classes and 78% for five classes. Guan and colleagues [24] conducted a study aimed at detecting the location of arm fractures using X-ray images. In this research, they focused on modifying a previously used CNN architecture to emphasize the normal convolution process. Pixel transformation preprocessing was applied to reduce noise and enhance brightness in the images. Using a feature pyramid architecture, features were extracted from the preprocessed images, and five feature maps at different scales were generated. These feature maps were used to identify regions of interest, resulting in a total of 256 regions of interest to determine the locations of fractures. Additionally, the detection area was expanded to detect small fractures. As a result, bounding boxes containing fractures were predicted using the obtained feature vectors. The dataset consisted of 4004 X-ray images, with training and test sets containing 3392 and 612 images, respectively. Expert radiologists were involved in drawing bounding boxes containing fractures. The performance of this study, as measured by the average precision criterion, was evaluated at 62.04%. Awan et al. [25] used a multi-scale guided attention-based context collection method to detect anterior cruciate ligament tears. The dataset used included 917 knee MRI images. As a result, 98.63% accuracy was achieved.

### 1.1. Motivation and Our Model

This study has emerged as a significant outcome of the notable advancements in the field of medicine, particularly in the diagnosis and treatment of musculoskeletal system radiographs. Presently, rapid developments in digitalization and image processing technologies in the field of radiology have enabled more precise and accurate analysis of musculoskeletal system radiographs. This dataset comprises a substantial collection of data obtained from Stanford Hospital, providing a foundation for research into the diagnosis and treatment of musculoskeletal disorders. This study aims to explore the potential of machine learning algorithms and artificial intelligence techniques in the analysis of such images using this valuable data source. To enhance the clinical applicability of the model, we have utilized the MURA dataset. This dataset includes radiographic images from various upper extremity regions such as the elbow, finger, forearm, hand, humerus, shoulder, and wrist. Furthermore, the results of this research may contribute to improved diagnoses and the development of new methods that can aid in the treatment of patients in clinical applications. Therefore, the motivation behind this study is to contribute to advancements in the diagnosis and treatment of musculoskeletal system radiographs and to fill the knowledge gap in this field.

### 1.2. Novelties and Contributions

In this section, we will present the novelties and contributions of our study.

The novelties of our research are as follows:

- We propose a fixed-size patch division method for extracting local features during the feature extraction phase. Unlike traditional methods, this new approach enables the more effective processing of images, resulting in the extraction of more prominent features.
- We conduct end-to-end training using only the images from the orthopedic MURA dataset, utilizing the suggested patch division method and a pre-trained EfficientB0 CNN model. This presents a distinct approach compared to previous studies and ensures effective results while preserving the uniqueness of the dataset.
- Deep features are generated using the retrained EfficientB0 CNN network. These features capture and render the significant information contained within the images for further analysis.
- Neighborhood component analysis (NCA) is employed, known for its ability to select the most informative features. Subsequently, classification results are obtained by deploying an SVM classifier using these selected features.
- We introduce a model incorporating deep features generated with our proposed patch division method. This model provides a deep feature engineering approach based on rectangular patch division, contributing to enhanced feature extraction.

The contributions of our model are as follows:

- We employ a novel approach to partitioning images into horizontal and vertical segments. This approach facilitates multi-level deep feature extraction and offers an effective means of extracting features located in the lower portions of the images.
- The deep feature extraction process is performed starting from the last fully connected layer of the EfficientB0 network, known for its efficient network architecture. This approach differs from prior studies and leads to more effective results.
- NCA is used to select the most distinctive features from the feature extraction results. This enables the model to utilize more precise and distinctive features, contributing to the improvement of results.

## 2. Material and Method

### 2.1. Dataset

**Figure 1:**
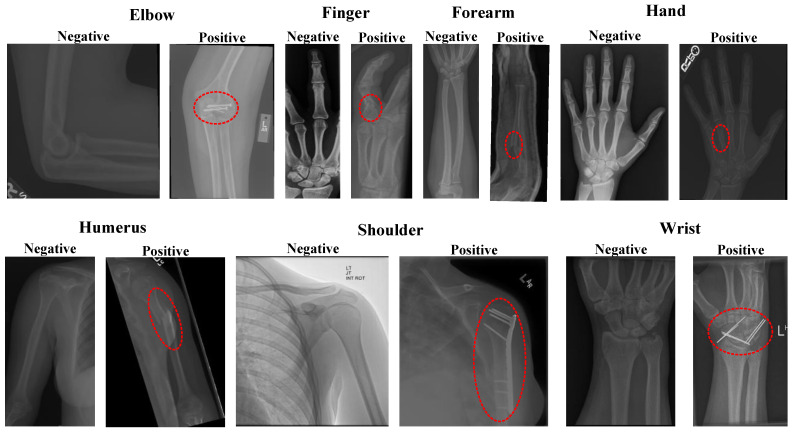
MURA dataset sample images. In the figure, abnormal images are circled in red.

The MURA dataset, which contains a sufficiently large dataset for anomaly detection in musculoskeletal radiographs, was created by the Stanford University Machine Learning Group [26]. It is noted that this dataset was manually labeled as normal/negative or abnormal/positive (fractured or with implants) by board-certified radiologists from Stanford Hospital between the years 2001 and 2012. The dataset includes images of seven standard upper extremities: elbow, finger, forearm, hand, humerus, shoulder, and wrist. Example images from the dataset are shown in Figure 1.

**Figure 2:**
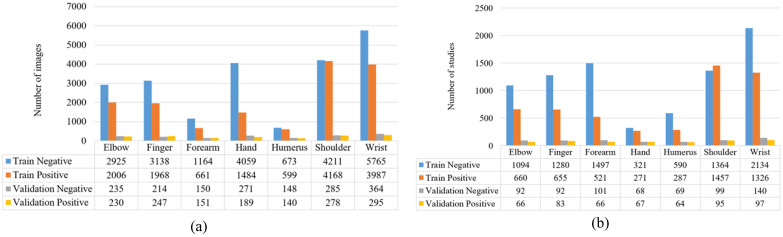
Numerical information for MURA dataset. (**a**) Distribution of the number of images in each class in the dataset. (**b**) Distribution of study numbers for each class in the dataset.

MURA is a musculoskeletal radiography dataset comprising data from 12,173 patients, 14,656 studies, and over 40,000 radiographic images. The distribution of the number of images and studies per class is illustrated in Figure 2.

### 2.2. Method

**Figure 3:**
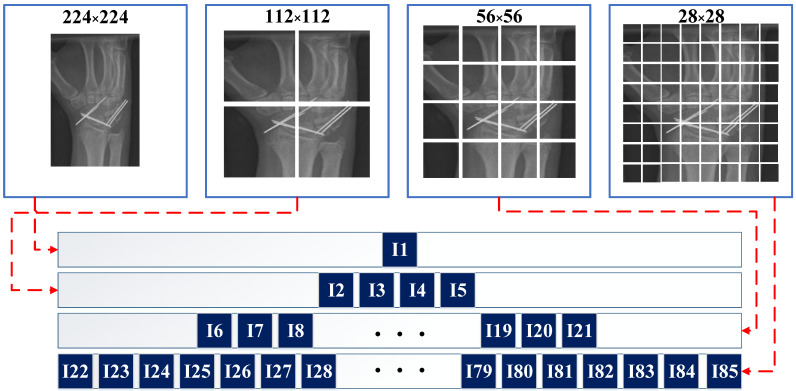
Splitting images into different sizes.

**Figure 4:**
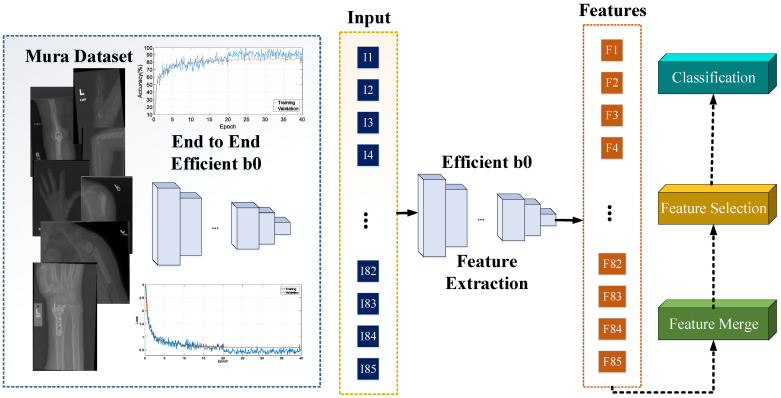
Schema of the proposed model. (See Figure 6 for accuracy and loss graphs).

In the scope of this study, we propose a new deep feature extraction architecture for classifying orthopedic images in the MURA dataset. We refer to this feature extraction structure as ‘pyramid deep feature extraction,’ and the schematic representation of this model can be observed in Figure 3. Our proposed model utilizes a pre-trained EfficientB0 CNN model that has been end-to-end trained with the MURA dataset. The primary objective of the model is to generate comprehensive features from orthopedic images. The block diagram of the proposed model is presented in Figure 4. As shown in Figure 3, orthopedic images from the colored MURA dataset were first resized to 224 × 224 dimensions.

During the resizing process, we utilized a bilinear interpolation method. As a result of this operation, the 224 × 224 sized image was divided into 64 segments of 28 × 28 dimensions. Subsequently, the retrained EfficientB0 CNN model was used for feature extraction on these 64 segments. For a MURA orthopedic image, 64 × 1000 features were extracted using EfficientB0. In the second step of the pyramid, the 224 × 224 RGB image was resized to dimensions 56 × 56, yielding 16 image segments. Feature extraction was performed on these 16 segments again using the EfficientB0 CNN model. For a MURA orthopedic image, 16 × 1000 features were extracted using EfficientB0. In the third step of the pyramid, the 224 × 224 RGB image was resized to dimensions 112 × 112, resulting in 4 image segments. From these 4 image segments, 4 × 1000 features were extracted using EfficientB0. In the fourth step of the pyramid, 1 × 1000 features were extracted from the original 224 × 224 RGB image using EfficientB0. The features obtained in the four stages of the pyramid were combined, resulting in a total of 85,000 features extracted for orthopedic images in the MURA dataset. The proposed pyramid model was applied to the EfficientB0 model. The concatenated features were selected using the NCA feature selection algorithm. Subsequently, the selected features were classified using 10-fold cross-validation and an SVM classifier.

The steps of the model are detailed below.

Here is a summary of the steps in your process:

Step 1: The EfficientB0 network was retrained using the images from the MURA dataset, which contained 40,005 images.

Step 2: The 224 × 224 RGB image was divided into 64 segments of 28 × 28. Each segment was resized back to 224 × 224. Using the MatMul fully connected layer of the retrained network, 64,000 features were extracted.

Step 3: The 224 × 224 RGB image was divided into 16 segments of 56 × 56 dimensions. Each segment was resized back to 224 × 224. Using the MatMul fully connected layer of the retrained network, 16,000 features were extracted.

Step 4: The 224 × 224 RGB image was divided into 4 segments of 112 × 112. Each segment was resized back to 224 × 224. Using the MatMul fully connected layer of the retrained network, 4000 features were extracted.

Step 5: Features obtained in Steps 2 to 4 were combined, resulting in a total of 85,000 features.

Step 6: From the 85,000 features obtained in Step 5, 1000 features were selected using the neighborhood component analysis (NCA) algorithm.

Step 7: The selected 1000 features were classified using a 10-fold cross-validation and an SVM classifier.

#### 2.2.1. Efficient b0

EfficientNet is a convolutional neural network architecture that introduces a novel scaling model using compound scaling coefficients [27]. Unlike conventional convolutional neural networks that scale network dimensions such as width, depth, and resolution randomly, in EfficientNet, each network dimension is scaled uniformly with a fixed scaling coefficient. The compound scaling method has been found to improve model accuracy and efficiency compared to traditional scaling methods. This method can determine that if the input image is large, more layers and channels are needed to detect finer details in the larger image.

The EfficientNet architecture is fundamentally based on the idea of a mobile inverted bottleneck convolution. EfficientNetB0 in particular is a revision of the EfficientNet network designed for mobile and embedded devices. EfficientNetB0 consists of 5.3 million parameters. In addition to the squeeze-and-excitation blocks, it also incorporates the inverted bottleneck residual blocks used in the MobileNetV2 network. EfficientNet’s architecture and scaling approach have shown significant improvements in performance across various computer vision tasks, making it a popular choice for deep learning applications, especially on resource-constrained platforms like mobile devices.

#### 2.2.2. Feature Selection

Neighborhood component analysis (NCA) [28] is a supervised learning algorithm employed in the classification process to select the most optimal features. This method aims to maximize separability among different classes, i.e., it endeavors to identify the features that best differentiate the classes from each other. The fundamental principle of NCA is to assess how effectively each feature contributes to the separation between classes. This evaluation is geared towards enhancing classification performance. In other words, NCA selects the attributes that most effectively highlight the differences between classes. The operational logic of NCA involves utilizing a feature vector for each item or data point and optimizing these vectors to enhance classification performance. These optimized vectors emphasize the features that assist in better distinguishing between classes. Consequently, NCA is an approach employed for feature selection in classification tasks, with its primary objective being the identification of attributes that optimize class separation and improve classification performance. As a result, it enables the attainment of superior classification outcomes.

#### 2.2.3. Classification

Support vector machines (SVMs) [29] represent a novel technique suitable for binary classification tasks that encompass parametric applied statistics, neural networks, and machine learning. SVMs are a potent method for constructing classifiers, with the aim of creating a decision boundary between two classes. They facilitate the prediction of labels for one or more feature vectors. The fundamental concept behind SVMs is to maximize the margin between the two classes, thereby obtaining a hyperplane. An SVM is a supervised learning method that generates input–output mapping functions from a set of labeled training data. SVM models are closely related to artificial neural networks, and an SVM utilizing a sigmoid kernel function is akin to a two-layer feedforward neural network. One of the key assumptions of SVMs is that all examples in the training set are independently and identically distributed [30]. SVMs can be applied to both classification and regression problems. In SVM regression, the core idea is to find a linear separator function that closely reflects the nature of the available training data, adhering to the principles of statistical learning theory.

## 3. Experimental Results

**Figure 5:**
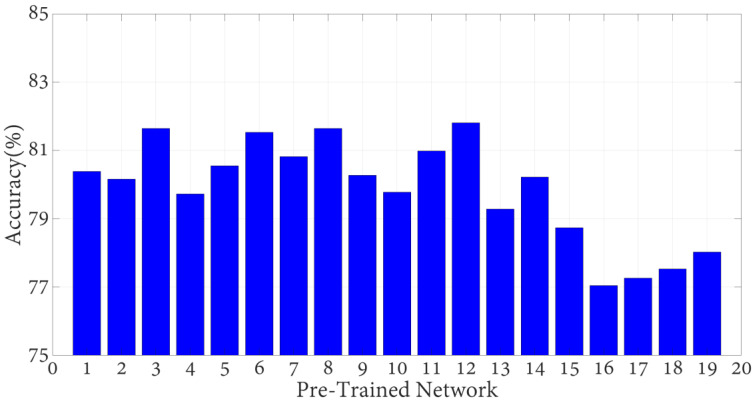
Accuracy results of 19 pre-trained models (Legened: 1: Resnet18, 2: Resnet50, 3: Resnet101, 4: Darknet19, 5: Mobilenetv2, 6: Darknet53, 7: Xception, 8: Densenet201, 9: Shufflenet, 10: Nasnetmobile, 11: Nasnetlarge, 12: Efficientnetb0, 13: Inceptionv3, 14: Inceptionresnetv2, 15: Googlenet, 16: Alexnet, 17: Vgg16, 18: Vgg19, 19: Squeezenet).

The effectiveness of the provided pyramid deep feature extraction method was validated using seven different upper extremity images (elbow, finger, forearm, hand, humerus, shoulder, and wrist) from the MURA dataset. This pyramid deep feature extraction model was implemented using MATLAB (R2023a) software. Below, the configuration of the desktop computer used for this process is provided. The PC utilized was equipped with a 3.0 GHz 13th generation Intel(R) Core(TM) i9 processor and 128 GB of RAM and ran on the Windows 11 Pro operating system. MATLAB (m) vfiles were employed to implement the recommended pyramid deep feature extraction and the NCA algorithm. The Matlab Classification Learner Toolbox was used for classifying the results and a quadratic support vector machine (SVM) classifier was employed to achieve the best classification results. In this study, we first conducted a feature extraction process using 19 different pre-trained models to determine the CNN (convolutional neural network) model that would be used to classify images in the wrist class. These 19 pre-trained models were as follows: Resnet18 [31], Resnet50 [31], Darknet19 [32], Mobilenetv2 [33], Darknet53 [32], Xception [34], Efficientnetb0 [27], Shufflenet [35], Nasnetmobile [36], Nasnetlarge [36], Densenet201 [37], Inceptionv3 [38], Inceptionresnetv2 [39], Googlenet [40], Alexnet [41], Vgg16 [42], Vgg19 [42], and Squeezenet [43]. We then classified the extracted features using an SVM (support vector machine) classifier. We evaluated the classification accuracy of each model and we present these results in Figure 5. This graph helped us understand which model performed best on the given dataset.

**Figure 6:**
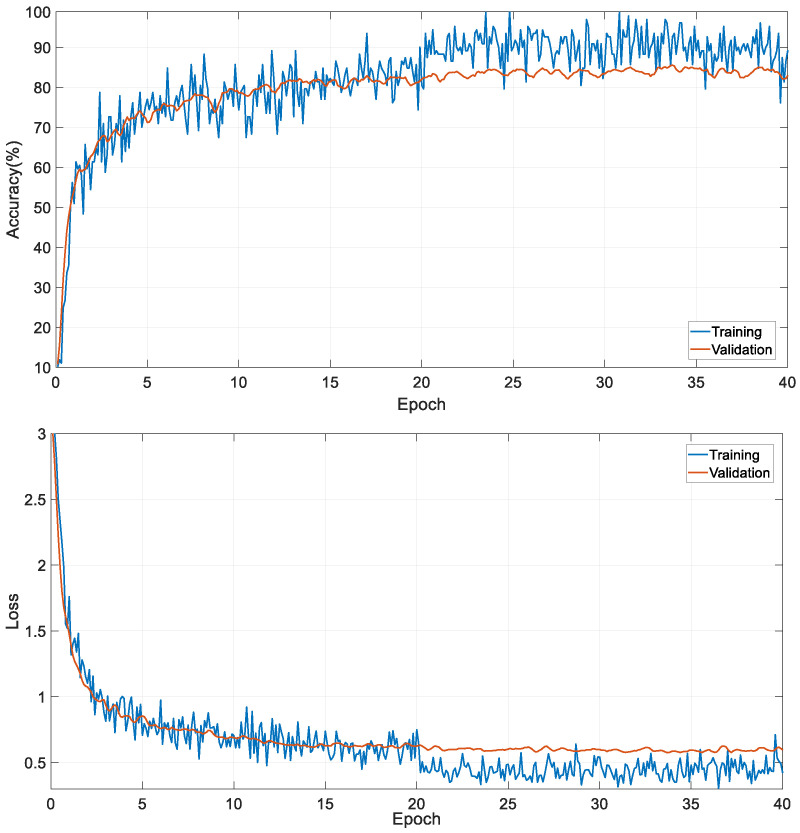
Training and validation curves of Efficientnetb0.

Due to the highest accuracy obtained among the results, we decided to use the Efficientnetb0 CNN network in our study. We conducted end-to-end training using the Efficientnetb0 CNN network on the 40,005 images from the MURA dataset, which consists of seven classes. The accuracy and loss curves obtained from this training are shown in Figure 6.

**Figure 7:**
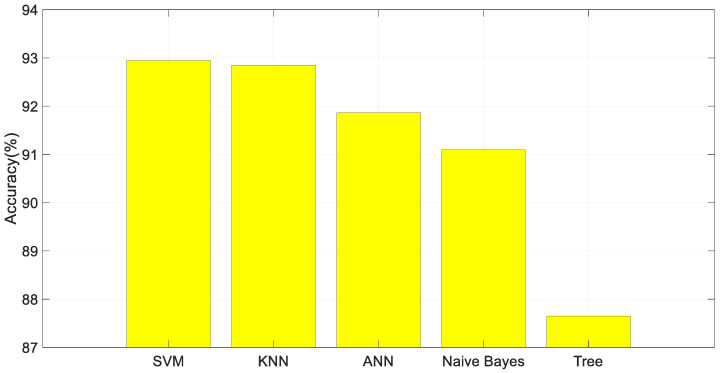
Classification results.

The performance results of different classifiers with the help of features obtained from wrist images with the proposed method are compared in Figure 7. The SVM classifier achieved higher accuracy than other classifiers.

**Figure 8:**
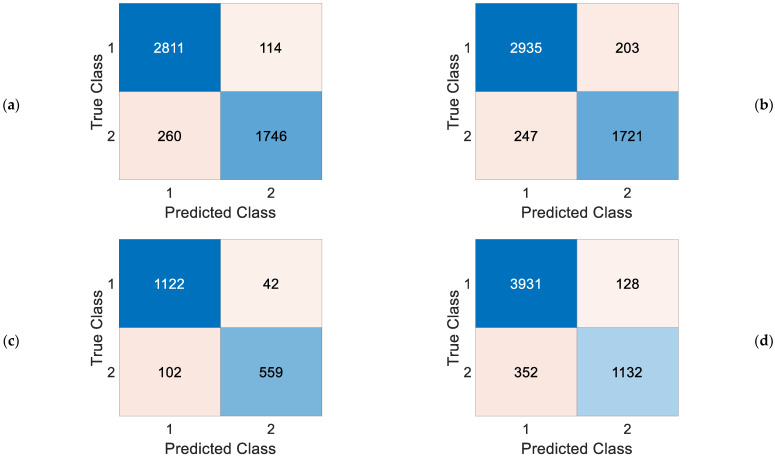

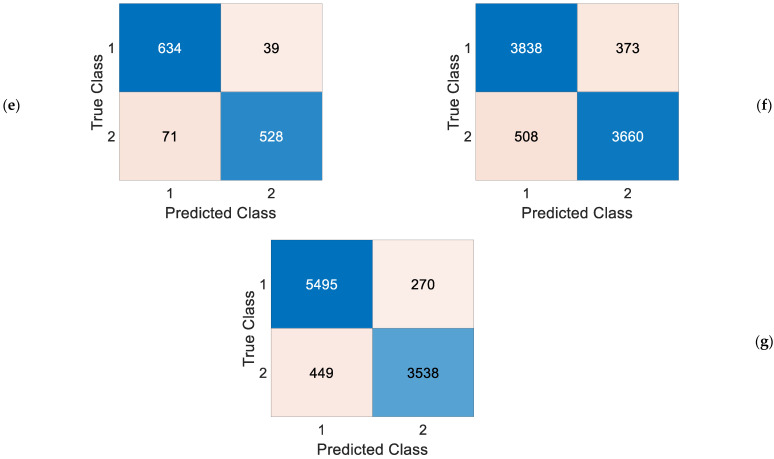
Confusion matrices: (**a**) elbow, (**b**) finger, (**c**) forearm, (**d**) hand, (**e**) humerus, (**f**) shoulder, (**g**) wrist. Legend (1 = negative, 2 = positive).

Subsequently, we used the end-to-end network we obtained to classify the images of the seven classes within the MURA dataset separately, using the recommended method. In this study, important performance metrics were employed to evaluate the success of a model or algorithm. These metrics were calculated using confusion matrices, as shown in Figure 8. Performance metric results for the entire dataset are tabulated in Table 1. Among the performance metrics, accuracy, F1 score, recall, and precision were included.

This article presents medical classification results for different body regions. The results are detailed with various performance metrics measured for both negative and positive classes across different regions such as the elbow, finger, forearm, hand, humerus, shoulder, and wrist regions. The results indicate that the model demonstrates success in accurately predicting positive results in regions with a high ability to do so (e.g., ‘wrist’ and ‘humerus’). Particularly, the ‘hand’ region is noted to have a lower capability in accurately predicting positive results, which could have significant implications in clinical applications. Therefore, it is recommended to enhance the model and make performance improvements specifically in the ‘hand’ region. These findings contribute to our understanding of the potential and limitations of deep learning methods in radiological diagnostic applications. Noteworthy aspects of these metrics include high recall values, which are effective in correctly classifying the positive class and can thus make significant contributions to clinical diagnosis and treatment planning.

**Figure 9:**
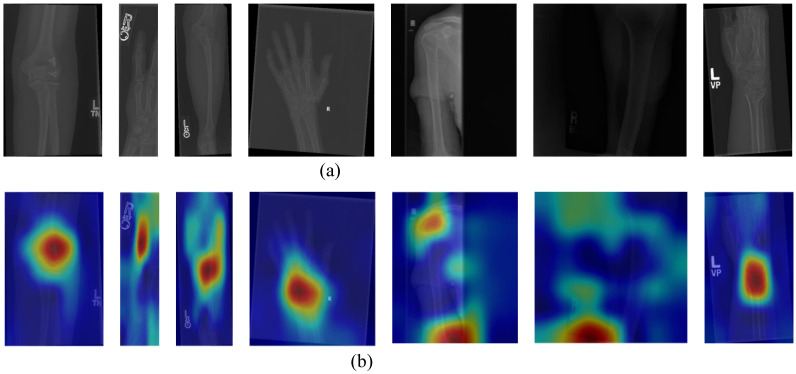
Hot-maps of the false predicted samples. (**a**) Original Images; (**b**) Hot-Maps.

We achieved a classification accuracy ranging from 89.49% to 92.63% by utilizing transfer learning-based models. To validate the effectiveness of our model, we adopted the technique of generating heatmaps as explanatory results. Among various approaches for obtaining such results, gradient-weighted class activation mapping (GradCAM) [44] stands out prominently [45]. To investigate instances where our model made incorrect predictions, we applied GradCAM to the relevant images, and one type of these instances is illustrated in Figure 9. Based on Figure 9, it is evident that our proposed model did not effectively focus on the region of interest (ROI). These images did not exhibit clear visual cues.

## 4. Discussion

This study has introduced a novel deep feature extraction model called pyramid deep feature extraction for the analysis of musculoskeletal radiographs. This model is a recommended approach for classifying orthopedic images in the MURA dataset. The proposed pyramid deep feature extraction model has proven to be successful in extracting effective features from orthopedic images. Enabling feature extraction from images of different dimensions provided an effective way to handle the diversity of the dataset. The obtained results demonstrate that this approach is an effective tool for orthopedic image classification (see Table 2).

Karthik et al. [46] proposed the MSDNet, which combines features of the AlexNet and ResNet CNN models, achieving an accuracy of 82.69%. However, they encountered lower performance in imbalanced classes such as hand and wrist in the MURA dataset they used in their study. Oh et al. [47] utilized 10 separate models for the identification and classification of fractures in wrist X-ray images. By incorporating HyperColumn-CBAM structures into the EfficientNet-B0 and DenseNet169 models, they achieved an accuracy of 87.50%. Lu et al. [48] developed a universal fracture detection system through deep CNN methods. Initially, image enhancement techniques were applied to enhance image quality. Subsequently, data augmentation was employed to expand the dataset’s scale. Eventually, the classification of fractured and healthy bones was performed using Ada-ResNeSt, achieving a mean precision of 68.4%. Liang et al. [49] proposed a novel multi-network architecture called MSCNN-GCN for the detection of musculoskeletal system abnormalities. This architecture conducts the detection of abnormalities through musculoskeletal radiographs, combining a multiscale convolutional neural network (MSCNN) with a fully connected graph convolution network (GCN). The study obtained the following F1 score results for different body regions: 79.20 for finger, 86.20 for humerus, 84.80 for elbow, 81.40 for forearm, 85.80 for hand, 85.70 for shoulder, and 96.80 for wrist. These results indicate the effectiveness of the proposed approach in detecting musculoskeletal abnormalities. In this study, we conducted a comprehensive evaluation of our proposed pyramid deep feature extraction model in terms of its utility for medical experts. As demonstrated in Table 2, our model exhibited superior performance compared to other models. These findings underscore the effectiveness of the pyramid deep feature extraction model in detecting images from the MURA dataset. Its high accuracy and performance make it a valuable tool in the domain of disease diagnosis and analysis.

### Testing the Proposed Pyramid Deep Feature Extraction Model with an Alternative Dataset

**Figure 10:**
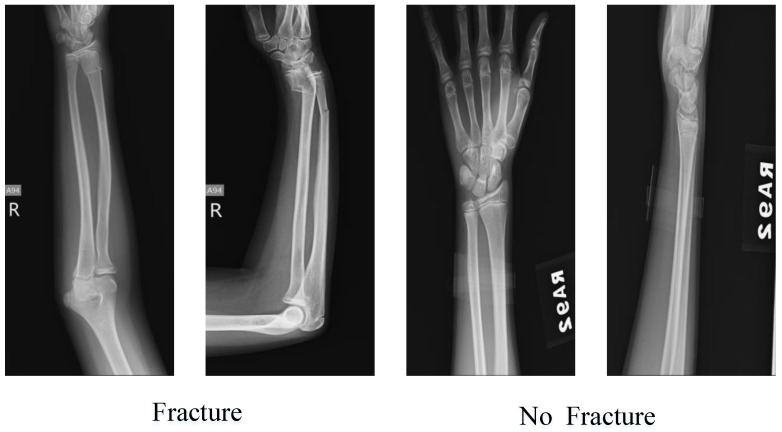
Kaggle dataset sample images.

**Figure 11:**
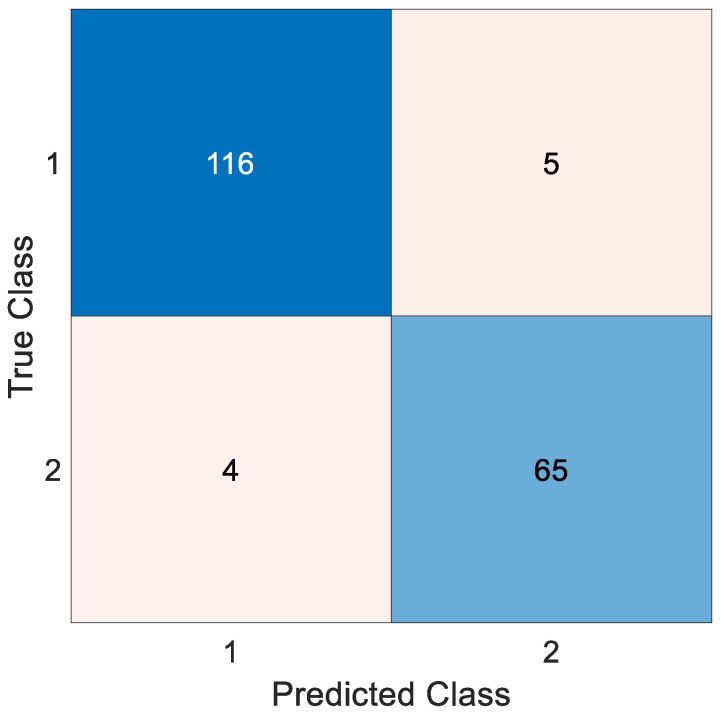
Matrix confusion for Kaggle dataset. (Legend 1: Fracture Present 0: Fracture absent).

In our study, we utilized the Kaggle dataset titled ‘Pediatric Radius Fracture’ [50] to assess the performance of the pyramid deep feature extraction model we proposed. This dataset consisted of two main classes: ‘fracture present’ and ‘fracture absent.’ The ‘fracture present’ class contained a total of 121 images, while the ‘fracture absent’ class comprised 69 images. Sample images from the dataset are illustrated in Figure 10. To evaluate the results obtained using our recommended approach, we employed a confusion matrix to demonstrate the model’s performance, as depicted in Figure 11.

In the scope of this study, we evaluated the performance of the pyramid deep feature extraction model, and the results we obtained were quite promising. In the classification process, our model achieved an accuracy rate of 95.26%, which reflects its high ability to accurately classify the dataset. Furthermore, the F1 score was calculated as 96.27%, indicating a balanced performance of the model in terms of both accuracy and precision. Notably, our model exhibited an impressive recall capability in addition to precision, with a recall rate of 95.87%, demonstrating its accurate classification of the ‘fracture present’ and ‘fracture absent’ classes. These findings indicate that our proposed method successfully diagnosed fractures in the dataset.

## 5. Conclusions

This study introduced and evaluated the pyramid deep feature extraction model for the automatic classification of musculoskeletal radiographs. The experiments conducted demonstrated that the proposed model can effectively classify various upper extremity images. Furthermore, we showed that the model can be successfully used to obtain customized classification results for different body regions. The end-to-end training process with the pre-trained Efficientb0 CNN model enabled the accurate classification of various upper extremity classes in the MURA dataset. This can be considered an important step in the automatic analysis of musculoskeletal radiographs for clinical applications. The results of this study include accuracy rates obtained for different upper extremity regions: 92.04% for elbow, 91.19% for finger, 92.11% for forearm, 91.34% for hand, 91.35% for humerus, 89.49% for shoulder, and 92.63% for wrist. These results demonstrate that the proposed model can successfully classify different upper extremity regions. This success highlights its potential as an assistive tool in the automatic analysis of musculoskeletal radiographs. Such automated analysis tools have the potential to expedite clinical diagnosis processes and provide healthcare professionals with more precise results. In conclusion, this study presents a deep feature extraction model that can be used for the automatic analysis of musculoskeletal radiographs. Future research should focus on further testing and refinement of this model in clinical applications. The integration of such technologies into medical diagnosis and treatment processes holds the potential to enhance healthcare services for patients. Additionally, it is anticipated that this study will be adapted to clinical practice in the future. To accomplish this, a multicenter dataset comprising a substantial number of diverse image classes will be assembled. Subsequently, an application suitable for clinical use will be developed. This will enable faster and more accurate disease diagnosis. The main limitation of our study is the instability of the number of images in the dataset used for training the developed models.

## Figures and Tables

**Table 1:** Performance metric results.

Classes		Accuracy (%)	Specificity (%)	F1-Score (%)	Recall (%)	Precision (%)
Elbow	Negative	92.04	86.18	93.45	96.10	90.94
	Positive		96.10	89.86	86.18	93.87
Finger	Negative	91.19	87.45	92.88	93.53	92.24
	Positive		93.53	88.44	87.45	89.45
Forearm	Negative	92.11	84.57	93.97	96.39	91.67
	Positive		96.39	88.59	84.57	93.01
Hand	Negative	91.34	76.28	94.25	96.85	91.78
	Positive		96.85	82.51	76.28	89.84
Humerus	Negative	91.35	88.15	92.02	94.21	89.93
	Positive		94.21	90.57	88.15	93.12
Shoulder	Negative	89.49	87.81	89.70	91.14	88.31
	Positive		91.14	89.26	87.81	90.75
Wrist	Negative	92.63	88.74	93.86	95.32	92.45
	Positive		95.32	90.78	88.74	92.91

**Table 2:** Comparison of our model with state-of-the-art methods.

Study	Method	The Results (%)
Karthik et al. [46]	MSDNet CNN (Alexnet + Resnet)	Accuracy: 82.69
Oh et al. [47]	HyperColumn-CBAM Model-DenseNet16	Accuracy: 87.50
Lu et al. [48]	Ada-ResNeSt101	Accuracy: 68.40
Liang et al. [49]	DenseNet169, CapsNet, MSCNN	F1-Score Results Finger: 79.20 Humerus: 86.20 Elbow: 84.80 Forearm: 81.40 Hand: 85.80 Shoulder: 85.70 Wrist: 96.80
Proposed model	Exemplar Efficient b0, NCA, SVM	Accuracy: Finger: 91.19 Humerus: 91.35 Elbow: 92.04 Forearm: 92.11 Hand: 91.34 Shoulder: 89.49 Wrist: 92.63

## Data Availability

In this paper, the dataset is publicly available.
